# Central European Vaccination Advisory Group (CEVAG) guidance statement on recommendations for influenza vaccination in children

**DOI:** 10.1186/1471-2334-10-168

**Published:** 2010-06-14

**Authors:** Vytautas Usonis, Ioana Anca, Francis André, Roman Chlibek, Inga Ivaskeviciene, Atanas Mangarov, Zsófia Mészner, Roman Prymula, Pavol Šimurka, Eda Tamm, Goran Tešović

**Affiliations:** 1 Vilnius University Clinic of Children's Diseases, Faculty of Medicine, Vilnius University, Vilnius, Lithuania; 2 University of Medicine and Pharmacy - Department of Pediatrics 'Prof. Dr. Alfred Rusescu' Institute for Mother and Child Care, Bucharest, Romania; 3 Chaussée de Huy, 136, 1325-Chaumont-Gistoux, Belgium; 4 Faculty of Military Health Sciences, University of Defense, Hradec Králové, Czech Republic; 5 Infectious Diseases Hospital, Sofia, Bulgaria; 6 National Institute of Child Health, Budapest, Hungary; 7 University Hospital Hradec Králové, University Hospital, Trencin, Slovakia; 8 Department of Pediatrics, University Hospital, Trencin, Slovakia; 9 Children's Clinic of Tartu University Hospital, Estonia; 10 Department for Pediatric Infectious Diseases, University Hospital for Infectious Diseases, Zagreb, Croatia

## Abstract

**Background:**

Influenza vaccination in infants and children with existing health complications is current practice in many countries, but healthy children are also susceptible to influenza, sometimes with complications. The under-recognised burden of disease in young children is greater than in elderly populations and the number of paediatric influenza cases reported does not reflect the actual frequency of influenza.

**Discussion:**

Vaccination of healthy children is not widespread in Europe despite clear demonstration of the benefits of vaccination in reducing the large health and economic burden of influenza. Universal vaccination of infants and children also provides indirect protection in other high-risk groups in the community. This paper contains the Central European Vaccination Advisory Group (CEVAG) guidance statement on recommendations for the vaccination of infants and children against influenza. The aim of CEVAG is to encourage the efficient and safe use of vaccines to prevent and control infectious diseases.

**Summary:**

CEVAG recommends the introduction of universal influenza vaccination for all children from the age of 6 months. Special attention is needed for children up to 60 months of age as they are at greatest risk. Individual countries should decide on how best to implement this recommendation based on their circumstances.

## Background

Influenza is a serious infectious disease that continues to contribute to significant morbidity and mortality worldwide. The WHO estimates up to five million cases annually with mortality between 250,000 and 500,000 in the industrialised world [1]. In Europe excess deaths due to influenza are estimated to be between 40,000 and 220,000 per season. Yearly influenza vaccination benefits have been demonstrated and vaccination for high-risk groups is well recognised in Europe and the rest of the world as a means of preventing infection and the complications that develop from influenza such as pneumonia [1-4]. High-risk groups include infants and children with existing health complications as well as the elderly (>65 years of age) and vaccination of these groups is current practice in many countries. Within the member states of the European Union there is an objective to achieve over 75% vaccination coverage in elderly populations. However, there are no such objectives in paediatric populations within Europe despite the evidence that healthy children are susceptible to influenza, sometimes with complications [5]. The burden of disease in infants is greater than in elderly populations [6,7] and rates of hospitalisation attributable to influenza are similar to those in older adults. To reflect this morbidity, universal influenza vaccination in all children from the age of 6 months up to 18 years is current practice in the USA and from 6 to 23 months in Canada [8,9]. Universal vaccination of healthy children is not widespread in Europe despite clear demonstration of the benefits of vaccination in reducing the large health and economic burden of influenza [10]. In Central Europe guidelines on paediatric influenza immunisation have not been made to date and the current report represents a summary of evidence and the formulation of advisory guidelines to help decision-making about paediatric influenza immunisation.

## Discussion

### The under-recognised burden of influenza in children

The burden of influenza in children is under-recognised [11-13]. The incidence of influenza in children under 5 years of age is far greater than in the elderly, with estimates showing an attack rate of 30% and respiratory illnesses peaking in the age group at 1-2 years [7]. Although overall influenza-associated mortality rates in children were not high, a US study showed 63% of the 153 reported influenza-associated paediatric deaths during the 2003-04 influenza season were less than 5 years of age [14]. In this study 33% had an underlying condition known to increase the risk of influenza-related complications and 20% had other chronic conditions; however, 47% had previously been healthy.

Children under 2 years of age are at highest risk of influenza and are most likely to develop serious complications such as pneumonia, secondary bacterial infection, and sepsis [15]. Excess hospitalisations are high in young children, particularly those younger than 12 months of age. Healthy children under one year of age are hospitalised for influenza-associated illnesses at similar rates to those for adults in high-risk groups [16]. The high rate of hospitalisation in children was largely responsible for introduction of universal vaccination in the USA. Despite high rates of hospitalisation, influenza cases are generally under-reported and it is likely that the number of notified cases does not reflect the real-life frequency of influenza. Influenza infections are frequently unrecognised clinically despite contributing to significant numbers of hospitalisation (annual rate 0.9 per 1000 children) [13].

The largest proportion of paediatric influenza-related illness is associated with the outpatient setting [5]. Outpatient visits associated with influenza in young children are 10- to 250-fold more common than hospitalised cases [13]. Children between the ages of 6 and 23 months have the highest rates of visits to clinics and emergency departments attributable to influenza [13]. These medically-attended influenza cases are also under-reported due to the potential for incorrect diagnosis and lack of laboratory confirmation. Laboratory confirmation is frequently not used to diagnose influenza in young children and rarely used in the outpatient setting. The diagnosis of influenza is usually made on the basis of patient history, clinical signs and symptoms and knowledge of the local epidemiological situation. Due to overlap of symptoms with other respiratory disease infection, influenza in children is easily misdiagnosed [13].

Typical symptoms of early influenza include mainly sudden high fever, cough and absence of rhinitis. Children do not always show these symptoms. Whilst high fever is a prominent sign of influenza, unlike adults, many children treated for influenza in the outpatient setting present with rhinitis during the early phase of the illness, making clinical distinction from a 'cold' difficult in this age group [17]. In young children the clinical presentations of influenza-related illness can consist of pneumonia, croup, bronchitis and sepsis, and these conditions might not be attributed to influenza if laboratory-confirmed data are not available [15]. Several rapid antigen detection tests are available and provide results in 10-30 min but with reduced sensitivity (70%-90% in children) compared with RT-PCR and with viral culture. Accuracy of these assays depends greatly on patient age, length of illness, sample type, and possibly viral type [18]. Our understanding of the clinical presentation of paediatric influenza is based only on studies examining hospitalised children with severe forms of influenza which may alter our perception concerning the signs and symptoms of influenza in children [17]. It has been shown that for children with laboratory-confirmed influenza, only 28% of hospitalised children and 17% of children seen in the clinic are correctly diagnosed by their treating physicians [13]. The accuracy of diagnosis is poor, particularly in the early and late phases of outbreaks [19]. Clinical diagnosis is poorest in children under 3 years of age, the group that has the greatest burden of disease from influenza. In this group a clinical diagnosis sensitivity of 21% and positive predictive value of 16% were observed [19]. One result of low diagnosis is that the number of cases reported does not reflect the actual frequency of influenza. The use of rapid testing for influenza may enhance the precision of the influenza diagnosis but the reduced sensitivity of these tests means that negative test results should be confirmed with RT-PCR and/or viral culture [18,20].

### Influenza vaccine

The efficacy and effectiveness of trivalent inactivated vaccine (TIV) in healthy children (under 19 years of age) has been examined in a number of meta-analyses, overall vaccine efficacy estimates were similar for laboratory-confirmed influenza (59-63%) and clinical cases (36-45%) [21-23]. Lower estimates for clinically diagnosed cases are possibly due to inclusion of misdiagnosed non-influenza cases and a proportion of cases that would not be prevented even with a totally efficacious vaccine [22]. All three meta-analysis studies highlight a lack of extensive data for children less than 2 years of age that preclude meaningful analysis.

For children aged less than 5 years the vaccine efficacy against influenza is broad ranging, from 12 to 83% [24]. The efficacy and effectiveness of influenza vaccinations depends on several factors, most importantly on the age and immunocompetence of the recipient, the similarity between the viruses in the vaccine and in circulation for a given influenza season, and the study design and outcome measured [8]. A recent case-control study over eight consecutive influenza seasons from 1999-2000 through 2006-07 has shown that TIV is highly effective in preventing laboratory-confirmed influenza in children <5 years of age when given as recommended by the American Academy of Pediatrics and the Advisory Committee on Immunization Practices (ACIP) of the Centers for Disease Control and Prevention (CDC), with effectiveness estimates of 86% [25].

Relatively few studies on influenza vaccine efficacy and effectiveness have been conducted in children aged between 6 and 23 months [26-28]. In one randomised clinical study in this age group, vaccine efficacy against culture-confirmed influenza was 66% during the 1999-2000 influenza season; however there was no significant reduction of culture-confirmed influenza during the 2000-01 season. It was noted that the attack rate was higher (15.9%) in the first season compared to the second (3.3%) [26]. In a retrospective cohort study in children 6 to 23 months of age, vaccine effectiveness was 25% against influenza-like illnesses and 49% against pneumonia or influenza during the 2003-04 influenza season [27]. In another retrospective study examining healthy children aged 6-21 months, vaccine effectiveness after two doses was estimated to be 87% against pneumonia or influenza-related office visits [28]. The influenza cases in both studies were not laboratory-confirmed.

The influenza vaccines currently approved for children and adults in Europe contain three strains of influenza viruses that are recommended annually by the WHO based on global surveillance. The vaccine contains one influenza A(H3N2) strain, one influenza A(H1N1) strain, and one influenza B strain and may change each year to reflect the most recent circulating forms of the virus. TIV is approved for use in children aged 6 months or more, as well as adults, and includes populations who have chronic medical conditions and women who are pregnant. There is currently no vaccine recommended for children less than 6 months of age.

An annual booster of influenza vaccine is recommended for all age groups. The TIV is injected into the deltoid muscle and does not cause influenza although minor side effects are noted. Inactivated influenza vaccines contain trace levels of egg protein so that individuals with allergies to egg protein should not receive the vaccine.

The inactivated influenza vaccine is safe and well-tolerated in children [29,30]. It very rarely causes immediate allergic reactions and there is no evidence of an increase in asthma exacerbations [31]. The risk of developing Guillain-Barré syndrome following influenza vaccination is at most one in 1,000,000 [8]. There is no evidence of any preventive effects of homeopathic supplements against influenza [32].

Influenza vaccines are the mainstay of efforts to reduce the substantial global health burden that influenza poses. In the USA influenza vaccination is recommended for all children six months of age or older, adults 50 years old or older, all individuals with chronic medical conditions and pregnant women, as well as people in contact with susceptible groups, such as health-care professionals. Given the global disease burden of influenza, the WHO has indicated that member states should evaluate the cost-effectiveness of introducing influenza vaccination into national immunisation programmes [33].

In Europe two doses of trivalent inactivated influenza vaccine are recommended in previously unvaccinated infants and children [34]. The two doses are administered at least one month apart for children aged 6 months to 8 or 9 years (depending on manufacturer's recommendations) who either receive an influenza vaccine for the first time or have not been exposed to influenza previously. Despite recommendations of multiple doses, young children often only receive one injection of influenza vaccine for primary vaccination. For children under 3 years of age a half dose is required per injection. For older children receiving the seasonal influenza vaccine for the first time, a single dose of the vaccine is appropriate. However, optimal dosage and scheduling in infants and children is currently not well established.

In the USA the number of influenza vaccine doses administered is age-dependent [8]. Children who are 9 years old or older who have not been vaccinated previously require one dose in their first season of immunisation. Children under the age of 9 years being vaccinated for the first time receive a second dose at least 4 weeks after the first. Children younger than 9 years of age who received only one dose of vaccine in the first season they were vaccinated should receive two doses of influenza vaccine the following season; this applies only to the influenza season that follows the first year that the child receives influenza vaccine.

In Canada children under 9 years of age who have never received an influenza vaccine require two doses administered 4 weeks apart [9]. Those who have been vaccinated with one dose in the previous year require two doses, while those who received two doses in the previous year would only require one dose. Children receiving vaccine for the third year or longer would only require one dose.

Before implementing any universal vaccination scheme it is vital to consider in addition to disease burden the safety of the vaccine to the recipient and to the whole population and the expectation of significant public health benefits with a demonstration of cost-effectiveness. The indirect benefit to the community of vaccinating healthy children, mostly of school age, has been evaluated in a systematic review and found to be potentially beneficial to the children and capable of generating significant health benefits and cost saving for the wider community [35].

In Europe the prevailing immunisation strategy is to prevent seasonal influenza primarily by protecting vulnerable (those at high risk) individuals as opposed to achieving herd immunity and reduce transmission in the community via universal vaccination. There is increasing support for immunising healthy children against influenza with the appreciation that children bear the vast burden of disease. Vaccination of children will protect against viral infection but will also protect against two common bacterial complications: otitis media and pneumonia. In addition to the direct benefit of reducing the burden of disease in children there is the indirect effect of reducing transmission by protecting unvaccinated adults against infection. Children are the main transmitters of the influenza virus during local outbreaks as they shed greater quantities of the virus and for longer periods of time than adults [12,36-38]. Universal vaccination of infants and children could result in decreased morbidity and mortality in other high-risk groups in the community [35].

As long ago as 1970 Monto *et al*. showed that indirect protection of adults was achieved by vaccinating schoolchildren [39]. In Japan the overall influenza-associated mortality rates were lowest during the winters of 1977-87 when influenza vaccination in Japanese schools was compulsory [40]. With around 60% of Japanese families consisting of grandparents also living in the same household, immunisation of all schoolchildren protected other at-risk members in their family. During the influenza epidemic of 1968-69 in the USA, in which 85% of schoolchildren had been vaccinated, overall respiratory illness rates dropped to one-third of the rates observed in an unvaccinated population [39] while vaccination of day care children (aged 24-60 months) reduced the incidence of febrile respiratory illnesses in unvaccinated family members by 42% [41]. Similar findings were reported in Italy where vaccination of children younger than 14 years significantly decreased the cases of respiratory infections and absenteeism from work among household contacts [42].

In addition to the community-wide protection against influenza provided by vaccinating children, there are potentially significant cost-related benefits. Indirect costs avoided include parental time costs, absence from work, loss of earnings and time costs among secondary cases. Parents lose an average of 1.3 working days caring for sick children and 0.4 days due to transmission of the infection [5]. A study in Finland showed that for every 100 influenza-infected children younger than 3 years old, 195 days of parental work were lost (mean duration 3.2 days) [11]. It is estimated that investing 1.7 million Euros in the vaccination of children <5 years of age will save 2.7 million Euros in health care costs [43]. Vaccination was found to be cost saving even with an assumed vaccine efficacy of 60% [43].

### Existing recommendations for vaccinating children against influenza

In 2006-07 an independent scientific panel convened by the European Centre for Disease Prevention and Control found that there were insufficient data to support widespread immunisation of children though the vaccines did induce immunity [34]. The review found considerable data from outside Europe but few data relevant to Europe itself and there was a lack of information on the European burden of disease in children. This view has recently been reconfirmed [44]. Although the evidence for immunisation of risk groups such as children and pregnant women is not strong for Europe, it should be noted that there is no evidence *against *immunisation of these groups [44]. The World Health Organization currently recommends yearly vaccination against seasonal influenza for children 6-23 months of age [45].

The number of countries adopting universal vaccination of infants and children in response to the burden of influenza in this age group is increasing. In Canada the National Advisory Committee on Immunization currently recommends influenza vaccination for all children aged 6-23 months of age [9]. ACIP has continuously updated its recommendations in the USA for influenza vaccination in children of 6-23 months of age (in 2003) to 6-59 months of age (in 2006) and finally extending their recommendation in 2009 to include all children and adolescents under the age of 19 years [8]. Interestingly, 48% of children received the first dose of influenza vaccine in the first year of the ACIP's recommendation for universal influenza vaccination [46]. This vaccination rate was higher than the vaccination rate among older children with high-risk medical conditions, for whom influenza vaccination has been recommended for many years. Universal age-based recommendations are more likely to be successful than risk-based. Health-care professionals and the public find it difficult to decide on eligibility using risk-based recommendations. Vaccination rates for those ≥65 years of age, for whom universal influenza vaccination is recommended, are consistently higher than among adults <65 years old with underlying high-risk conditions [47].

A 2008 survey of the 27 EU member states together with Norway and Iceland revealed that all countries recommended seasonal influenza vaccination in the period 2006-07 for older age groups (22 countries for those aged >65 years) [10]. In Austria the vaccine was recommended for all age groups. In Poland the recommendation is from the age of 50 whilst in Hungary immunisation is recommended from the age of 55. All countries recommended vaccinating high-risk patients and this includes any person irrespective of age with chronic pulmonary and cardiovascular diseases, with haematological or metabolic disorders, immunological disorders and renal disease, as well as residents of long-term care facilities. Within Europe, six countries, in addition to Austria, advise routine vaccination of children. Estonia recommends vaccination of all healthy individuals from the age of 6 months whilst Finland recommends vaccination of healthy children between 1 and 3 years. Latvia, Romania and Slovenia recommend vaccination between 6 months and 2 years whilst Slovakia has a broader age range from 6 months to 5 years for vaccination of healthy children. Whilst these countries recommend vaccination for children, a recent study suggested that the scientific evidence for immunising children in Europe was not strong but acknowledged that there was no evidence against immunisation of this group [44]. Table 1 summarises the current recommendations for vaccination of children according to country.

**Table 1:** Countries with recommendations to vaccinate healthy children according to age

Country	Age range
**North America**	
Canada [9]	6-23 months
USA [8]	6 months - 18 years
Europe*	
Austria	≥ 6 months
Estonia	≥ 6 months
Finland	1-3 years
Latvia	6 months - 2 years
Romania	6 months - 2 years
Slovakia	6 months - 5 years
Slovenia	6 months - 2 years

*Adapted from National Seasonal Influenza Vaccination Survey in Europe [56] and information provided on the Vaccinnet Romania website [57]

### CEVAG

CEVAG consists of regional experts from ten Central European countries: Bulgaria, Croatia, the Czech Republic, Estonia, Hungary, Lithuania, Poland, Romania, Slovakia and Turkey. The aim of CEVAG is to encourage the efficient and safe use of vaccines to prevent, control and if possible eradicate infectious diseases, by raising awareness of immunisation and by the compilation and distribution of appropriate information.

### CEVAG guidance statement

CEVAG recommends the introduction of universal influenza vaccination of all children, including healthy children and those with existing health complications, from the age of 6 months. Special attention is needed for children up to 60 months of age as they are at greatest risk. Individual countries should decide on how best to implement this recommendation based on their circumstances. The CEVAG guidance statement on recommendations for paediatric influenza immunisation is described in Table 2.

**Table 2:** Summary of CEVAG guidance statement on recommendations for influenza vaccination of all children

Consideration	CEVAG recommendation
Primary target group for influenza vaccination	Children
Age of vaccination	From 6 months of age, with special attention to the 6-60 month age group
Method of vaccine delivery	To fit within the existing vaccination schedule in the first and second year of life
Vaccination routine	Annual vaccination. For the first year of vaccination, all children should receive two doses with minimum interval of 4 weeks according to the manufacturer's recommendations. In the second year, children should receive a single dose of seasonal vaccine.
	For children aged between 6 and 35 months 0.25 mL doses are administered, equivalent to half the adult dose
Commonly reported reactions following vaccination	Mild soreness, redness, and swelling at injection site, fever, and aches. These symptoms usually begin soon after the injection, and last 1-2 days
Safety concerns	No safety concerns have been identified with the use of trivalent inactivated influenza vaccines with the exception that individuals with allergy to egg products should avoid immunisation

### Strategies for the future

Constant review of new immunisation strategies to protect against childhood influenza will be required in Europe and the rest of the world. The recent spread of 2009 Influenza A(H1N1) illustrates the speed with which new forms of the influenza virus spread around the globe and the threat from A/H5N1 remains high. Although the trivalent inactivated vaccine is the mainstay for paediatric and adult vaccination, other vaccines have been investigated. Most data are available on the live, attenuated, cold adapted intranasal vaccine (LAIV) that is licensed in the USA for children aged above 24 months and for adults aged 18-49 years. It is not licensed outside the USA. Recently published data from clinical trials suggest that LAIV has a higher relative efficacy compared with TIV in children <5 years of age [48-50]. In children aged <24 months one study suggested increased risk of wheezing and increased risk of hospitalisation in children aged between 6 and 11 months [49]. A subsequent smaller study did not find any evidence for these adverse events [50]. Another strategy for the future is the use of enhanced adjuvants in association with inactivated influenza vaccine.

The ability of an influenza vaccine to trigger an immune response against drifted viruses not in the vaccine formulation is of potential clinical benefit in very young children naive to influenza virus exposure. Antigenic drift of influenza viruses requires frequent adaptation of vaccines to accommodate this drift. One of the major challenges faced is to predict the drifts ahead of influenza seasons. Adjuvanted vaccines such as those using MF59 induce significantly higher immune responses against mismatched H3N2 and H1N1 strains than the licensed split vaccine, they have acceptable tolerability and a greater immunogenicity in young children compared with conventional split vaccines [51]. Recent development of a vaccine against the pandemic influenza A (H1N1) containing the adjuvant AS03 show high immunogenicity and are well tolerated in children aged 6 to 35 months [52].

Influenza vaccines need to provide sustained protection against illness as well as being effective. Vaccination with LAIV provides prolonged protection against influenza in children between 6 and 18 years of age that lasts 5-12 months after the vaccination is first administered. Multiple studies have shown that LAIV provides sustained protection against influenza caused by antigenically similar strains through a second influenza season in children aged 6 months to 18 years without the need to revaccinate [53,54]. No such protection data exist for adult populations.

The introduction of universal influenza vaccination for all children is clearly desirable but continued education for health-care professionals and the public on the benefits of immunisation and paediatric influenza is likely to increase acceptance of vaccination. A stronger motivation on the part of health-care professionals would also enhance the vaccination of children. Vaccine uptake in children across 11 European countries ranged from 4.2% in Ireland to 19.3% in Germany with two countries of the CEVAG region, the Czech Republic and Poland, showing intermediate rates of 7.6 and 9.2% respectively [55]. There is little doubt that increased awareness will overcome many of the barriers to vaccination.

## Summary

•The under-recognised burden of disease in children under 5 years is far greater than in elderly populations

•The number of paediatric influenza cases reported does not reflect the actual frequency of influenza with clinical diagnosis poorest in children under 3 years of age

•Universal vaccination of infants and children could result in decreased morbidity and mortality in other high-risk groups in the community

•CEVAG recommends the introduction of universal influenza vaccination for all children from the age of 6 months. Special attention is needed for children up to 60 months of age as they are at greatest risk. Individual countries should decide on how best to implement this recommendation based on their circumstances.

## Competing interests

VU has been the principal investigator in clinical studies supported by GlaxoSmithKline (GSK), Novartis, and Wyeth-Lederle Vaccines. He has also been a scientific consultant to Aventis Pasteur, Baxter, GSK, Merck, and Wyeth-Lederle Vaccines and has received sponsorship from these companies to attend scientific meetings. IA has been the principal investigator in clinical studies supported by GSK. She has also been a scientific consultant to GSK and Wyeth and has received sponsorship from these companies to attend scientific meetings. RC has been the principal investigator in clinical studies supported by GSK, Novartis. He has also been a scientific consultant to Baxter, GSK, Novartis, Aventis Pasteur and Pfizer and received sponsorship from the GSK and Aventis Pasteur to attend scientific meetings. II has been a scientific consultant to Baxter, GSK, Merck, and Wyeth-Lederle Vaccines and has received sponsorship from these companies to attend scientific meetings. AM has been a scientific consultant to Wyeth, GSK, Aventis Pasteur, Solvey Pharma and has received sponsorship from these companies to attend scientific meetings. RP is a member of advisory boards for GSK, MSD, Wyeth, Baxter, Aventis Pasteur and has received research grants and honoraria from GSK, Wyeth, Baxter, Aventis Pasteur, Novartis. PS has received consulting fees and lecture fees from GSK, Wyeth and Merck Sharp & Dohme (MSD). ET has received sponsorship from the GSK and PharmaSwiss to attend scientific meetings. GT has received sponsorship from GSK, MSD and Wyeth to attend scientific meetings. FA and ZM have no competing interests.

## Authors' contributions

RP highlighted the need for such recommendations for Central Europe that were discussed by all authors at the CEVAG meeting in May 2009. VU conceived the outline and produced the first draft of the manuscript. All authors were actively involved in the selection and review of all content and had full editorial control during the writing of the manuscript. All authors read and approved the final manuscript.

## Pre-publication history

The pre-publication history for this paper can be accessed here:

http://www.biomedcentral.com/1471-2334/10/168/prepub
